# Supplementary material for: Johari-Goldstein relaxation in quenched and irradiated chalcogenide glasses
Source: Newton. 2026 Mar 2;2(3):None. doi: 10.1016/j.newton.2025.100338 (PMC12953220; doi:10.1016/j.newton.2025.100338)
Supplement: Document S1. Figures S1–S5 and Notes S1–S4 [file mmc1.pdf]

NEWTON, Volume *2*

Supplemental information

Johari-Goldstein relaxation in quenched and irradiated chalcogenide glasses

Jacopo Baglioni, Alessandro Martinelli, Peihao Sun, Francesco Dallari, Lara Piemontese, Muhammad Umair, Fabian Westermeier, Michael Sprung, and Giulio Monaco

Note S1 Evaluation of the enthalpy variation in quenched glasses

The enthalpy variation induced by the annealing of thermally quenched glasses is calculated from the thermograms as shown in Fig. S1. The enthalpy is calculated integrating the difference between the Maier-Kelley extrapolation (dashed blue line) of the low-temperature values of the thermogram and the calorimetric traces [1].

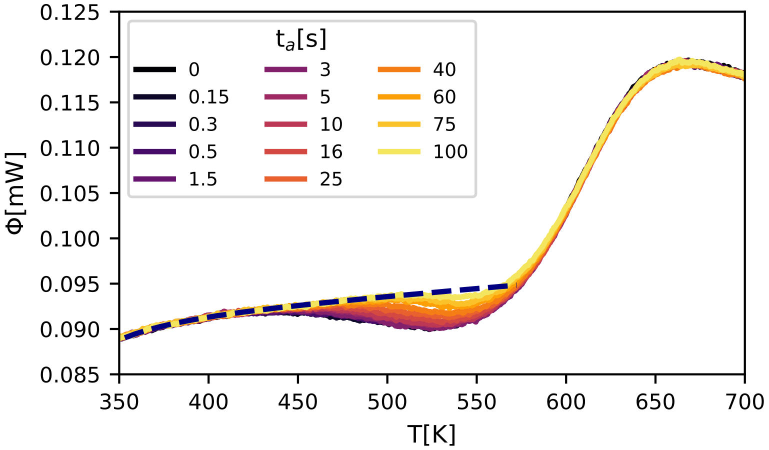

Figure S1: Themograms of a $GeSe_3$ glass quenched at 20 000 K s^{-1} and annealed at 368 K for various time intervals as listed in the legend. The dashed line is the Maier-Kelley extrapolation of the low temperature values of the thermal power.

Note S2 Secondary relaxation strength

Different strategies to separate the contribution of the primary and secondary relaxations in the calorimetric traces can be adopted, the one used in this work is shown in Fig. S2. The differential thermogram across the glass transition region is fitted with a Gaussian curve (black line) and the area below the curve is associated to the structural relaxation, while the remaining enthalpy contribution is assigned to the Johari-Goldstein (JG) relaxation. This method avoids the introduction of a threshold temperature between the two relaxations, thereby reducing the arbitrariness of their separation.

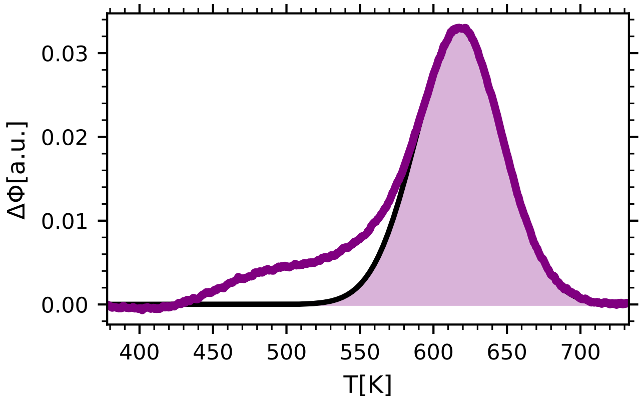

Figure S2: Differential thermogram of an irradiated $GeSe_3$ glass (purple line) and the Gaussian fit to the main peak (black line). The shaded region is proportional to the enthalpy contribution associated with the structural relaxation, while the residual area is associated with the Johari-Goldstein one.

Note S3 Stretched exponential fit to the enthalpy relaxation data

The values of the relaxation time, τ_a, and shape parameter, β_a, obtained from the fit of a stretched exponential function, Eq. 2 in the main text, to the enthalpy relaxation data as a function of the annealing time reported in Fig. 3 and Fig. 4 of the main text are shown in Fig. S3 and Fig. S4 for the thermally quenched and for the irradiated $GeSe_3$ glasses, respectively. The dashed line in panel **A** of Fig. S3 is the best fitting Arrhenius function, Eq. 3 in the main text, to the enthalpy relaxation data. The results obtained from the fit are discussed in the main text. The Arrhenius function with the same parameters is also reported in panel **a** of Fig. S4 to demonstrate compatibility with the enthalpy relaxation data for the irradiated glasses. For what concerns the shape parameters, they show no clear dependence on temperature, and have the average values β_a=1.06 ± 0.01 (0.63 ± 0.08) for the quenched (irradiated) glasses. The lower value of β_a for the irradiated glasses is compatible with the theoretical idea that a broad range of relaxation times in an heterogeneous structure is induced by irradiation [2].

Note S4 Thermograms at different probing rates

The thermograms collected as a function of the probing rate are reported in Fig. S5 **A**. They refer to a glass quenched from the melt to room temperature at $10\,\mathrm{K\,s^{-1}}$, irradiated for 420 s and then probed at various heating rates, r,

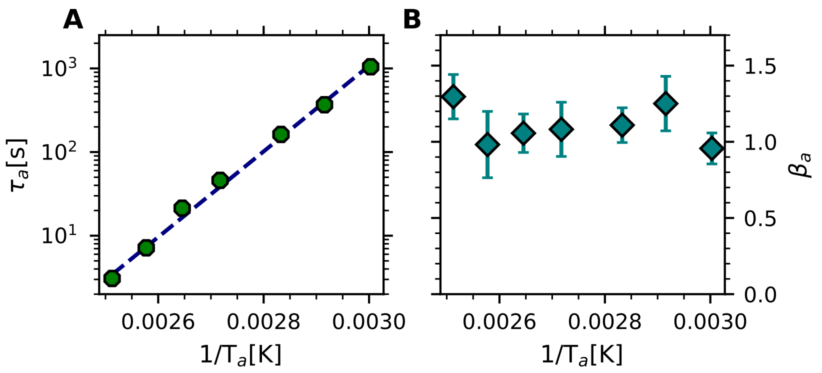

Figure S3: Relaxation time (**A**) and shape factor (**B**) obtained from fitting a stretched exponential function to the enthalpy relaxation data in Fig. 3 in the main text. The dashed line is the best fitting Arrhenius function used to extract the activation energy of the Johari-Goldstein relaxation.

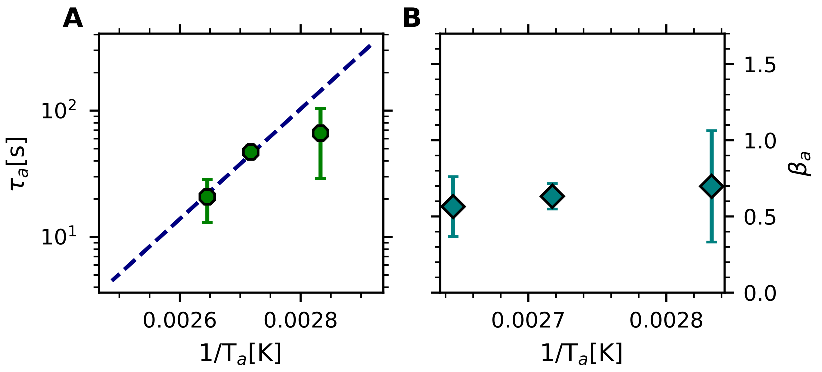

Figure S4: Relaxation time (**A**) and shape factor (**B**) obtained from fitting a stretched exponential function to the enthalpy relaxation data in Fig. 4 in the main text. The dashed line is the Arrhenius function obtained from fitting the enthalpy relaxation data for the quenched glasses and reported in Fig. S3.

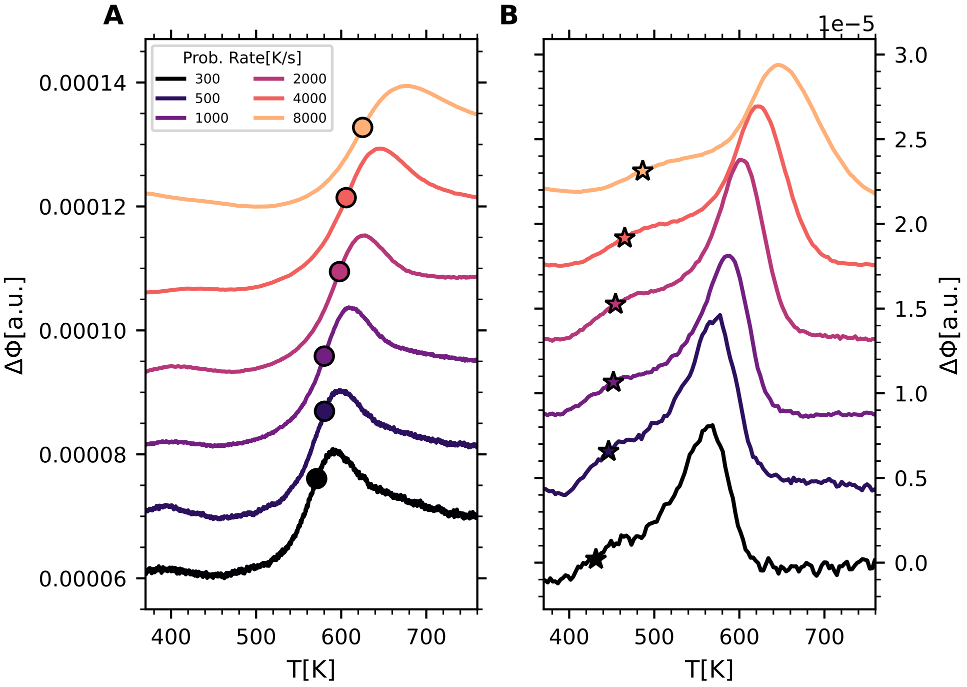

Figure S5: **A** Thermograms measured for $GeSe_3$ glasses irradiated for 100 s at room temperature and probed with different heating rates r (listed in the legend). The curves have been normalized by the absolute value of the rate. The circles mark the points of the maximum derivative of the enthalpy recovery peak. **B** Differential thermograms of the data in **A** computed with reference to the trace of a glass quenched at $10\,\mathrm{K\,s^{-1}}$ and then probed at the rate r. The stars mark the position of the maximum derivative of the exothermic signal associated with the JG relaxation.

ranging from $300\,\mathrm{K\,s^{-1}}$ to $8000\,\mathrm{K\,s^{-1}}$ and listed in the legend. For each probing rate, a differential trace was computed using the trace of a glass quenched at $10\,\mathrm{K\,s^{-1}}$ and then directly probed at the rate r. The obtained differential traces are reported in Fig. S5 **B** and are the same as in Fig. 5 in the main text. The characteristic temperatures of the enthalpy recovery peak (circles, associated to the structural relaxation) and of the exothermic peak appearing in the differential thermograms (stars, associated with the JG relaxation) are also reported in Fig. S5 **A** and **B**, respectively. These characteristic temperatures, which have been used in Fig. 6 in the main text in combination with the probing rates, correspond to the maximum value of the derivative of the enthalpy recovery peak (circles in Fig. S5 **A**) and to the maximum value of the derivative of the exothermic signal in the differential thermograms (stars in Fig. S5 **B**).

Supplemental References

[1] Y.Z. Yue, J.deC. Christiansen, and S.L. Jensen. Determination of the fictive temperature for a hyperquenched glass. *Chemical Physics Letters*, 357(1):20–24, 2002.

[2] Vassiliy Lubchenko and Peter G. Wolynes. Photon activation of glassy dynamics: A mechanism for photoinduced fluidization, aging, and information storage in amorphous materials. *The Journal of Physical Chemistry B*, 124(38):8434–8453, August 2020.
