# Supplementary material for: Johari-Goldstein relaxation in quenched and irradiated chalcogenide glasses
Source: Newton. 2026 Mar 2;2(3):None. doi: 10.1016/j.newton.2025.100338 (PMC12953220; doi:10.1016/j.newton.2025.100338)
Supplement: Document S2. Article plus supplemental information [file mmc2.pdf]

Newton

Article

Johari-Goldstein relaxation in quenched and irradiated chalcogenide glasses

Graphical abstract

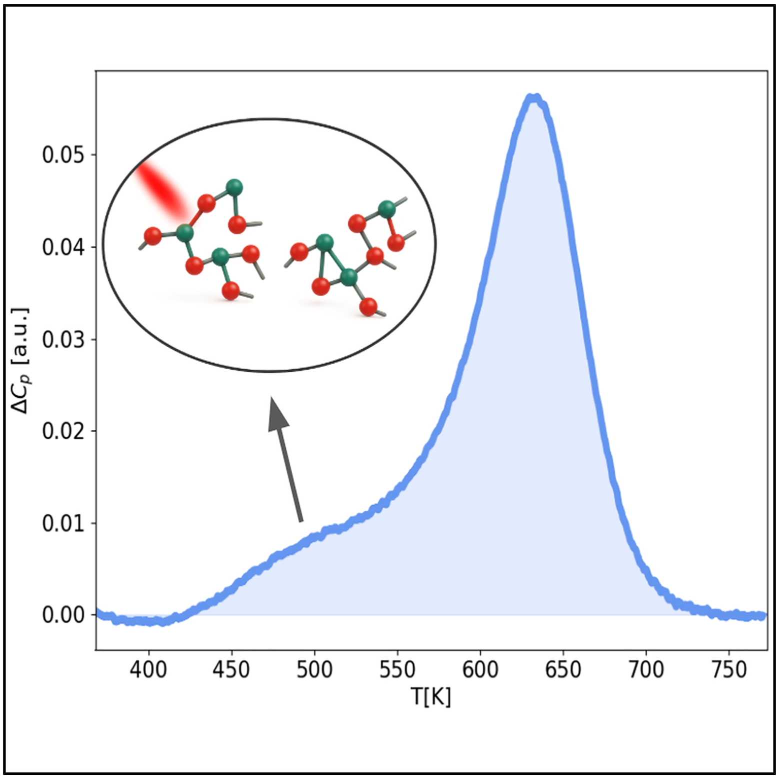

Highlights

- High-enthalpy glasses are produced by thermal quenching and X-ray irradiation
- Both methods increase the strength of the secondary Johari-Goldstein relaxation
- Iso-enthalpic glasses produced by different methods are different glasses
- The secondary relaxation originates from defect regions within the glass network

Authors

Jacopo Baglioni, Alessandro Martinelli, Peihao Sun, ..., Fabian Westermeier, Michael Sprung, Giulio Monaco

Correspondence

jacopo.baglioni@unipd.it (J.B.), giulio.monaco@unipd.it (G.M.)

In brief

The study of relaxation processes in glasses reveals information on mechanisms governing phenomena such as crystallization. Baglioni et al. show that X-ray irradiation increases the enthalpy of chalcogenide glasses, amplifying the secondary Johari-Goldstein relaxation and reflecting enhanced defect-induced heterogeneity. These results establish X-ray irradiation as a precise tool to control structural heterogeneity and mechanical behavior, providing a versatile approach to designing glasses with tunable relaxation dynamics and tailored mechanical properties.

Baglioni et al., 2026, Newton 2, 100338
March 2, 2026 © 2025 The Author(s). Published by Elsevier Inc.
https://doi.org/10.1016/j.newton.2025.100338

CellPress

Newton

CellPress
OPEN ACCESS

Article

Johari-Goldstein relaxation in quenched and irradiated chalcogenide glasses

Jacopo Baglioni,[1,5,*] Alessandro Martinelli,[1,3] Peihao Sun,[1] Francesco Dallari,[1] Lara Piemontese,[1] Muhammad Umair,[1] Fabian Westermeier,[2] Michael Sprung,[2] and Giulio Monaco[1,4,*]
[1]Department of Physics and Astronomy "Galileo Galilei", University of Padova, Via F. Marzolo 8, 35131 Padova, Italy
[2]Deutsches Elektronen-Synchrotron DESY, Notkestraße 85, 22607 Hamburg, Germany
[3]Present address: Laboratoire Charles Coulomb, Université de Montpellier, CNRS, 34095 Montpellier, France
[4]Senior author
[5]Lead contact
*Correspondence: jacopo.baglioni@unipd.it (J.B.), giulio.monaco@unipd.it (G.M.)
https://doi.org/10.1016/j.newton.2025.100338

ACCESSIBLE OVERVIEW Understanding relaxation processes in glasses is essential for linking their microscopic dynamics to macroscopic mechanical behavior. As a liquid is cooled toward the glass-transition temperature (T_g), its relaxation dynamics separate into two distinct processes: the structural (α) relaxation, involving large-scale cooperative rearrangements, and the faster Johari-Goldstein (JG) or β relaxation, a localized motion that persists below T_g. The JG relaxation governs the residual mobility of glasses and plays a central role in phenomena such as plastic flow and crystallization. Here, the JG relaxation is investigated in high-enthalpy $GeSe_3$ glasses using fast-scanning calorimetry. High-enthalpy glassy states are reached through two independent routes: rapid thermal quenching from the melt and X-ray irradiation of the solid glass. Both methods increase structural disorder and reveal the JG relaxation as an exothermic signal below T_g in the calorimetric traces, corresponding to the release of stored enthalpy from defect regions. Although rapidly quenched and X-ray-irradiated samples can exhibit comparable enthalpy levels, their calorimetric traces differ in shape, indicating distinct microscopic pathways to high-enthalpy states. Notably, the strength of the JG relaxation is higher in irradiated glasses than in isenthalpic glasses produced by thermal procedures, although this difference is reduced for long-irradiated glasses. These results support the view that the secondary relaxation originates from defect or loosely connected regions within the glass network. Such regions act as elementary plastic units whose number increases on increasing the enthalpy of the glass until the material yields. This work establishes X-ray irradiation as a controllable and versatile method, complementary to melt quenching, for tuning structural heterogeneity and thereby tailoring the mechanical and relaxation properties of glasses.

SUMMARY

When a liquid is cooled down to temperatures close to the glass transition, the relaxation dynamics are characterized by two timescales associated with the structural relaxation and a secondary process known as the Johari-Goldstein (JG) or slow β relaxation. The JG relaxation is related to many crucial properties of glasses, such as their plastic response, and is here investigated using fast-scanning calorimetry in high-enthalpy $GeSe_3$ glasses. High-enthalpy states are reached by two methods: (1) increasing the cooling rate used to quench the melt and (2) irradiating the glass with X-rays. Both methods make the JG relaxation visible in the calorimetric traces, where it appears as an exothermic signal at temperatures below the glass transition. The JG relaxation can be associated with mobile regions produced by quenching or defect regions produced by irradiation. These findings strongly support a general connection between the JG relaxation and local defect regions in the glass network and offer a new strategy to control via X-ray irradiation a key feature of the glass transition and, thereby, the related mechanical properties of the glass.

 © 2025 The Author(s). Published by Elsevier Inc.
This is an open access article under the CC BY license (http://creativecommons.org/licenses/by/4.0/).

INTRODUCTION

A glass is an out-of-equilibrium material. Its properties depend not only on the thermodynamic state variables, such as its pressure and volume, but also on the protocol used to prepare it.[1] Many applications of glasses, for example those in optics, require the optimization of their transparency and homogeneity, and to this aim glasses are typically annealed (aged) at temperatures below the glass-transition temperature, T_g.[2] Other applications, e.g., for bendable covers of cell phones, require improved ductility, which can be achieved by rejuvenating the glass,[3,4] i.e., bringing it into a higher-enthalpy state.

A glass can be brought to a higher-enthalpy state by different methods, including increasing the cooling rate used to quench it from the melt, plastic deformation,[5] and exposing it to radiation or particle beams.[6,7] These methods differ in their effectiveness, with extreme levels of enthalpy being obtained by constrained loading in compression.[3] The process clearly affects the microscopic structure and dynamics, where the modifications of macroscopic properties are eventually rooted.

Recent studies[8–10] demonstrate that a microscopic observable particularly affected by the enthalpy level of the glass is the Johari-Goldstein (JG) relaxation, also known as slow β[11,12] or secondary relaxation, where the primary relaxation, also known as structural or α relaxation, is responsible for the structural arrest of the undercooled liquid at T_g.[11] The JG process has been traditionally investigated using dielectric spectroscopy (DS) in organic glasses,[11,13] where it appears as a distinct peak, a shoulder, or even a tail of the structural relaxation, depending on its strength. More recently, and particularly in metallic glasses, it has also been investigated in detail via dynamical mechanical analysis (DMA)[14] and differential scanning calorimetry (DSC).[15,16] Notably, differently from spectroscopic techniques working in the linear response regime like DS and DMA, DSC probes relaxations indirectly, through the kinetics with which the thermodynamic state changes during the temperature scan,[17] and thus offers a different viewpoint of them. This specificity has proven to be particularly effective in investigating the JG relaxation in strong glasses, where most techniques fail to detect it due to its small strength. The strength of the JG relaxation can in fact be significant in some glasses and weaker in others.[18,19] For a given material, moreover, a decrease in strength has been reported for well-aged and more stable glasses[9] and an increase in strength for faster-quenched glasses.[16] The JG relaxation is the only fundamental relaxation active below T_g in the glass state, governing the residual mobility in the glass and important properties such as plasticity and crystallization propensity.[14,20,21] The connection between mechanical properties and the JG relaxation is particularly relevant. At least in organic[22] and metallic[23] glasses, the JG process is stronger in ductile than in brittle glasses. This relationship is further supported by the observation that shear transformation zones[24]—soft spots in the amorphous matrix, analogous to dislocations in crystals and related to the plastic response of glasses—share the same activation energy as the JG relaxation.[14]

Despite its relevance, the microscopic origin of the JG relaxation is still matter of active research. At the present stage, different conceptual frameworks—not obviously connected to each other—are used to describe it. For example, it can be described as a quasi-localized process occurring in loosely connected regions of the glass structure also known as "islands of mobility"[11] and, in this real-space picture, it can be related to the dynamic heterogeneities that characterize undercooled liquids and glasses.[25] Recently, the JG process has been associated with the percolation of these mobile regions[26] that has in turn been proposed to coexist with percolation of the "immobile regions" related to the structural relaxation within a double percolation scenario.[27,28] Another framework often used to describe the JG relaxation is the potential energy landscape (PEL),[29] where the JG relaxation can be associated with transitions between neighboring minima with small energy barriers, while the system explores large "metabasins" composed of several basins during the structural relaxation.[30] This topographic hierarchy in the PEL has emerged clearly in a recent numerical simulation study of an asymmetric dimer system, where the JG relaxation is related to rotational dynamics[31]; however, it remains to be demonstrated in systems with spherical interactions or in network glasses like the ones studied in this work.

We have recently investigated the effects of irradiating network glasses with X-rays,[32–34] demonstrating that prolonged irradiation rejuvenates these glasses toward a unique state that corresponds to the glass at the yield limit, and that the extent of rejuvenation is strongly influenced by the quenching rate used to prepare the sample.[34] Here, we aim at characterizing the JG relaxation, presenting a detailed comparative study performed by fast differential scanning calorimetry (FDSC) of $GeSe_3$ glasses in high-enthalpy states. These states are reached using two approaches: increasing the cooling rate for vitrification from the melt or irradiating a rather stable sample with hard X-rays until it yields. Both approaches induce, in the calorimetric traces, an exothermic contribution that appears at temperatures lower than T_g and that can be associated with the JG relaxation. In particular, we show that the JG relaxation is activated by X-ray irradiation and accompanies the sample's enthalpy increase until the yield point. However, there are also interesting differences between the JG processes induced by these two methods, as discussed hereafter.

RESULTS

High-enthalpy glasses by fast quenching and X-ray irradiation

First, we compare in Figure 1 the thermograms obtained using a fast calorimeter for isochemical $GeSe_3$ glasses quenched at different rates (Figure 1A) with the thermograms of a glass of the same composition quenched at 10 K s^{-1} and subsequently exposed to hard X-rays during different irradiation times (Figure 1C) (see methods for more details). The measured power has been normalized to its low-temperature value to facilitate comparison among the different samples. All traces shown in Figure 1 were collected on heating at 2,000 K s^{-1}. A clear endothermic signal, sometimes referred to as the enthalpy-recovery peak, appears above T_g for the most stable glasses (produced at low quenching rates or irradiated for short time intervals). This signal gradually decreases as the quenching rate/irradiation

CellPress
OPEN ACCESS

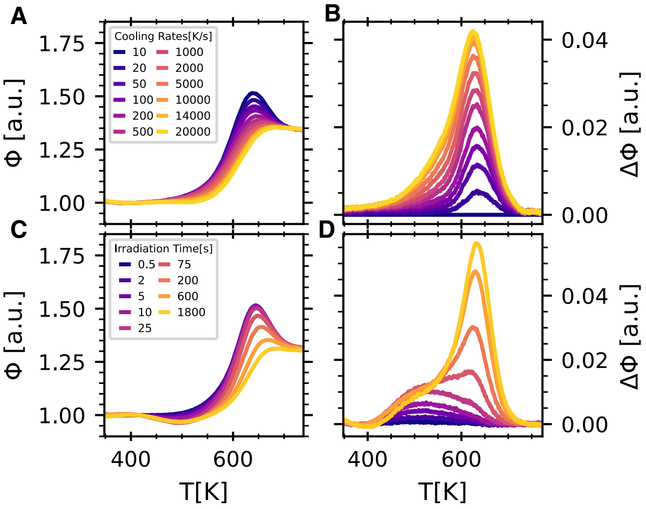

Figure 1. FDSC traces of $GeSe_3$ glasses

(A) Normalized thermograms (endo up) for glasses quenched at different rates (see legend).

(B) Difference between the reference thermogram (glass quenched at 10 K s^{-1}) and those reported in (A).

(C) Normalized thermograms of the reference glass exposed to X-rays for different time intervals (see legend).

(D) Difference between the reference thermogram and those of the irradiated glasses.

time increases. The extent of this endothermic peak depends on both the glass and the probing rate and is a signature of structural relaxation. More details can be observed in the differential traces computed with reference to a $GeSe_3$ glass quenched at 10 K s^{-1} and reported in Figure 1B for glasses prepared at different quenching rates and in Figure 1D for glasses irradiated for varying times. For the thermally prepared glasses, the differences occur mainly in proximity of the enthalpy recovery peak, reflecting variations in structural relaxation due to the quenching process. The differential signal for the irradiated glasses exhibits a distinct pattern. In fact, enthalpic variations are mainly located in the vicinity of the recovery peak only when the irradiation time is $\sim 10^2$ s or longer. In the short-irradiation-time (low-dose) regime, a wide peak appears in the differential thermograms at lower temperatures than those characteristic of the enthalpy recovery peak, and it persists in the trace also when, on increasing the dose, changes in the enthalpy recovery peak appear and grow. Signal at comparable temperatures is also present for the thermally quenched glasses, but only as a tail of the main peak.

To quantify the thermodynamic variations in the quenching and irradiation processes, we analyzed the FDSC traces by computing first the enthalpy changes of the glass as a function of quenching rate/irradiation time, i.e., computing the integral of the difference of the reference trace and those corresponding to different quenching rates/irradiation times. This difference in enthalpy, $\Delta\mathcal{H}$, is given by

$$\Delta\mathcal{H} = \int_{T_1}^{T_2} \left[c_{p,\mathrm{ref}}(T) - c_p(T)\right] \mathrm{d}T, \qquad \text{(Equation 1)}$$

where $c_p(T)$ ($c_{p,\mathrm{ref}}(T)$) is the specific heat for the quenched/irradiated glass (reference glass), and $T_1 = 380$ K ($T_2 = 700$ K) is in the glass below any relaxation (in the supercooled liquid region). The obtained values of $\Delta\mathcal{H}$ are reported, in absolute units, as violet circles in Figures 2A and 2B for the quenched and irradiated glasses, respectively (see methods for more details).

The shape of the differential calorimetric traces shows a tail-and-peak or shoulder-and-peak structure that has previously been observed in some glasses,[35] although, to the best of our knowledge, not for the case of irradiated glasses. The high-temperature peak in the differential traces provides information on the deformation, with respect to the reference thermogram, of the enthalpy recovery peak related to the structural relaxation for glasses prepared with different quenching rates/irradiation times. The low-temperature feature appears as a broad peak in the irradiated glasses and as a tail in the quenched ones. This feature has been observed in other glasses and is related to the JG relaxation process[15,36] (see also later discussion). It is therefore tempting to separate the two contributions in the differential traces. Although there is no unique way of doing this, the two contributions were here separated, fitting the enthalpy recovery peak with a Gaussian profile, thus obtaining the corresponding strength (see Figure S2 for more details). The remainder of the area under the differential trace is associated instead with the excess wing/shoulder contribution. While a different separation method might alter the absolute values of the two contributions, it would not influence any of the results discussed below. The results for the thus derived strengths are reported in Figures 2A and 2B for the quenched and irradiated glasses, respectively. In quenched glasses, we observe a strong correlation between the peak and tail contributions, both of which grow almost exponentially with increasing quenching rate. The situation is different for irradiated glasses: in this case, the lower-temperature contribution (shoulder) first grows to a plateau level in $\sim$100 s before the second contribution (enthalpy recovery peak) starts to change, in a two-step process.

Remarkably, the absolute total enthalpy of the irradiated glasses, as recently discussed in Baglioni et al.,[34] approaches a limiting value after a sufficiently long irradiation that we have associated with the yield point[33,34] and that corresponds, in $GeSe_3$ glasses, to an enthalpy level comparable to that reached by quenching at $\sim 10^4$ K s^{-1}, which is close to the maximum rate that can be reached with the used calorimeter. Instead, no sign of saturation can be observed in thermally produced glasses.

Given the qualitative differences among glasses prepared with the two different methods used, it is interesting to compare the calorimetric traces of iso-enthalpic glasses. A set of differential thermograms for glasses with a comparable total enthalpy is shown in Figures 2C–2F. The differences between the shapes of the differential calorimetric traces tend to disappear for higher-enthalpy glasses and are more prominent when the absolute value of the enthalpic variation with respect to the chosen reference is small, i.e., for low quenching rates and short irradiation times. In these cases, while for thermally quenched glasses the differential thermograms are dominated by a signal related to the enthalpy recovery peak, for the iso-enthalpic irradiated glasses the signal is rather centered at a lower temperature.

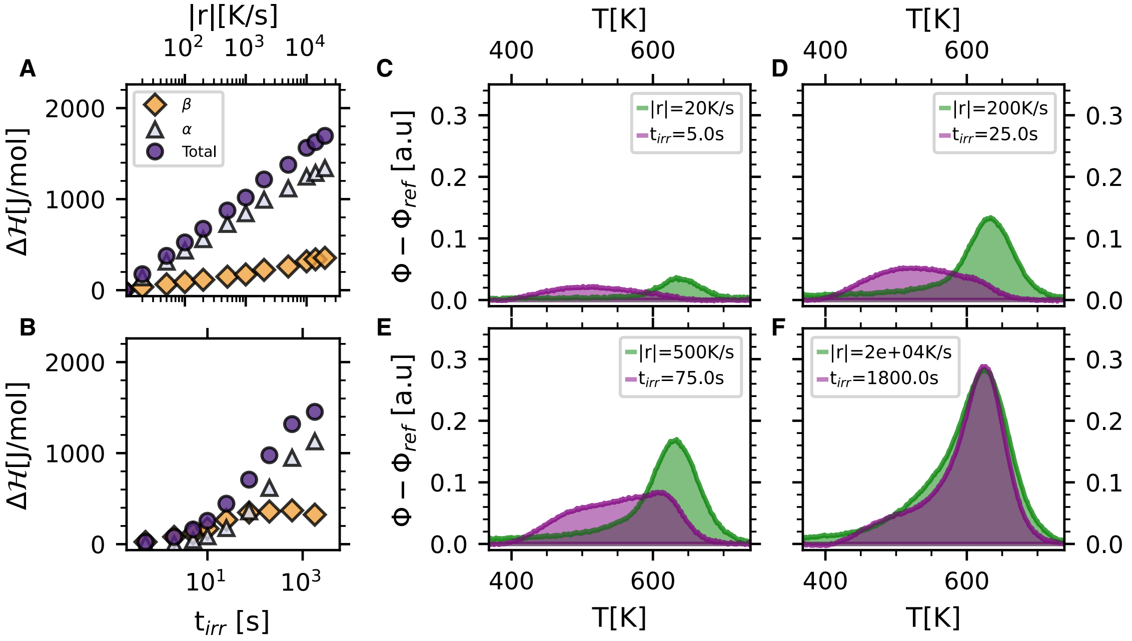

Figure 2. $GeSe_3$ glasses produced with different quenching rates and irradiation times

(A and B) Enthalpy variation (filled circles) of $GeSe_3$ glasses as a function of the quenching rate (A) and irradiation time (B). The contribution of the low-temperature, exothermic process to the total enthalpy variation is also reported for both series (orange diamonds) together with the contribution related to the enthalpy recovery peak (light-blue triangles).

(C–F) Differential thermograms of irradiated (violet) and thermally quenched (green) glasses; the corresponding quenching rate and irradiation time are reported within each panel. The glasses in each panel are (almost) iso-enthalpic, within 20% of the average enthalpy.

In other terms, starting from the very same glass obtained by quenching the melt at 10 K s^{-1}, different pathways can be taken using quenching rate or irradiation time to eventually reach the same high-enthalpy state. These results underline that a single parameter such as the enthalpy level is not sufficient to unequivocally describe the glass.[37] In the following, with three different experiments, we further investigate the differential thermograms associated with the two sets of glasses, demonstrating that, despite their differences, a single relaxation mechanism is at play there.

Calorimetric signature of the JG relaxation process

Calorimetry is a powerful technique to study relaxation processes in glasses. For instance, the exothermic signal observed in some hyperquenched glasses below the glass transition and similar to that observed here has been associated with the JG relaxation.[15,36] Here, we extend that approach to our high-enthalpy glasses performing a low-temperature annealing of the exothermic tail/shoulder to quantify the associated activation energy. The main idea behind this experiment is to first slow down the motions associated with the JG relaxation, which have relatively short relaxation times, by rapidly cooling the glass. Next, the glass is annealed for a duration on the order of the JG relaxation time (but short enough to prevent structural relaxation) and, eventually, the calorimetric trace is measured at a rate fast enough that no additional relaxation takes place.

The effect of annealing a glass that was initially quenched to room temperature at 20,000 K s^{-1} and then immediately heated to the annealing temperature T_a is illustrated in Figures 3A and 3B, which show the thermograms and the differential thermograms, respectively, measured heating at a rate of 2,000 K s^{-1} for a sequence of annealing times at T_a = 368 K. In fact, the bimodal structure (tail and peak) of the thermogram can be leveraged to selectively anneal the tail component for appropriate values of T_a. For each value of T_a in the range 333–398 K, the low-temperature tail appearing in the differential thermograms is progressively erased by annealing, as quantified by the enthalpy reduction as a function of the annealing time reported in Figure 3C. The time dependence of the enthalpy corresponding to this exothermic signal appearing below the glass transition, $\Delta\mathcal{H}_{ex}$, can be fitted, for each investigated T_a, with a stretched exponential of the form

$$\Delta\mathcal{H}_{ex} = \Delta\mathcal{H}_{tot} \exp\left[-(t/\tau_a)^{\beta_a}\right], \quad \text{(Equation 2)}$$

where $\Delta\mathcal{H}_{tot}$ is the initial enthalpy difference associated with the exothermic signal of the quenched glass. Figure 3C shows that the relaxation time, τ_a, of this enthalpy contribution decreases with increasing temperature. The results obtained for τ_a will be discussed in what follows together with the results of further experiments to be introduced hereafter, while those for the stretching coefficient, β_a, are reported in Figure S3.

We used the same approach to characterize the low-temperature exothermic shoulder that appears in irradiated glasses

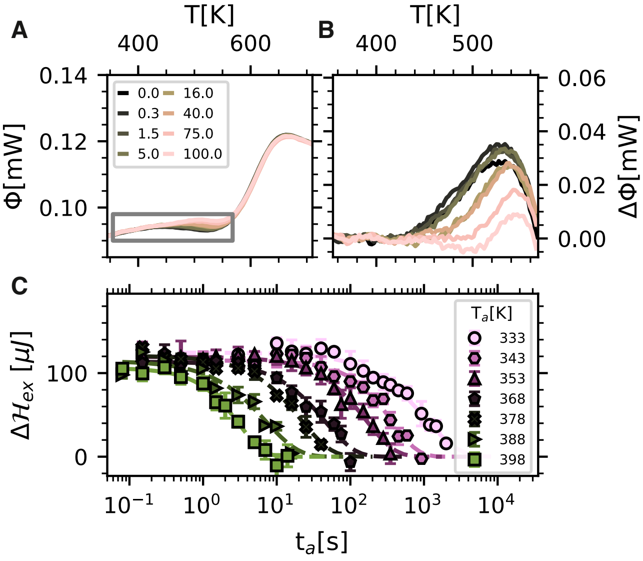

Figure 3. Effect of annealing on quenched glasses

(A) Thermograms of a glass quenched to room temperature at 20,000 K s^{-1} and annealed at T_a = 368 K for the annealing times t_a (s) reported in the legend.

(B) Differential thermograms between a common reference obtained extrapolating from low-T and the calorimetric traces reported in (A); for more details, see Figure S1. These differential traces are reported in the temperature range highlighted by the gray rectangle in (A).

(C) Enthalpy change corresponding to the exothermic low-temperature signal is reported as a function of annealing time for a series of annealing temperatures, as reported in the legend. The error bars represent standard deviation of the data. The lines through the experimental points are the best-fitting stretched exponential curves.

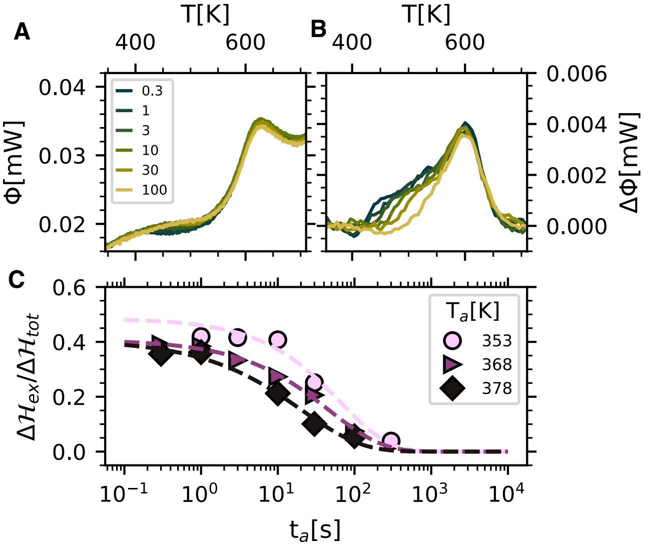

Figure 4. Effect of annealing on irradiated glasses

(A) Thermograms of a glass quenched at 10 K s^{-1}, irradiated at room temperature for 150 s, and annealed at T_a = 368 K for the annealing times t_a (s) reported in the legend.

(B) Differential thermograms between the trace of the reference glass and those reported in (A) (glasses irradiated and annealed).

(C) Enthalpy corresponding to the (progressively annealed) exothermic signal and normalized to the total initial enthalpy difference is reported as a function of annealing time for a series of temperatures, as reported in the legend. The lines through the experimental points are the best-fitting stretched exponential curves.

quantifying, for different values of T_a, the corresponding enthalpy reduction as a function of annealing time. In particular, we studied a glass quenched at 10 K s^{-1}, irradiated at room temperature for 150 s, heated up to T_a at 5,000 K s^{-1}, and probed at 2,000 K s^{-1} after an annealing time t_a at T_a. The thermograms and the differential thermograms between the initial glass (the one irradiated but not annealed) and those for the glasses irradiated and annealed are reported for T_a = 368 K in Figures 4A and 4B, respectively. Differently from the data reported in Figure 3B, the differential traces in Figure 4B are characterized by a contribution associated with the enthalpy recovery peak and a lower-temperature shoulder that is progressively erased by annealing. The presence of the first contribution is due to the fact that, as shown in Figure 1, an irradiation time of 150 s induces a small reduction of the enthalpy recovery peak, consistent with what is shown in Figure 4B. This contribution is unchanged upon annealing at the T_a temperatures selected here. The low-temperature shoulder appearing in the differential thermograms is instead progressively erased by the annealing process, and the corresponding reduction of enthalpy is reported as a function of time in Figure 4C for three different temperatures. This reduction in enthalpy is here reported normalized to the total initial enthalpy difference, $\Delta\mathcal{H}_{tot}$, which also includes the previously discussed change of the enthalpy recovery peak. Also in this case, this enthalpy reduction can be described by the functional form of Equation 2.

Figure 4C shows, as for the quenched glasses, a relaxation time, τ_a, which decreases with increasing temperature. The results obtained for τ_a will be discussed together with the ones obtained for the quenched glasses in the next paragraph, while those for the stretching coefficient, β_a, are reported in Figure S4.

We additionally performed a third experiment to further explore the origin of the exothermic signal that appears below the glass transition in the irradiated glasses. If this signal is related to a relaxation process, the maximum appearing in the differential thermograms should shift to a higher temperature as the probing rate increases. To this end, we prepared a glass quenching the melt to room temperature at 10 K s^{-1}, irradiated it for 100 s, and probed it at various heating rates r, ranging from 300 K s^{-1} to 8,000 K s^{-1}. For each probing rate, a differential trace was computed using the trace of a glass quenched at 10 K s^{-1} and then directly probed at the rate r. The thus obtained differential traces show clear changes, as reported in Figure 5. As the probing rate increases, both the low- and high-temperature components shift toward higher temperatures and broaden, similarly to what was recently reported in metallic glasses.[16] As already mentioned, the low-temperature contribution is absent in the trace of the reference glass (differently from the enthalpy recovery peak) and, therefore, the differential traces only serve to enhance its visibility. Fitting this low-temperature contribution using a Gaussian profile, it is possible to extract the temperature, T_{md}, at which its slope is maximum (see Figure S5 for more details).

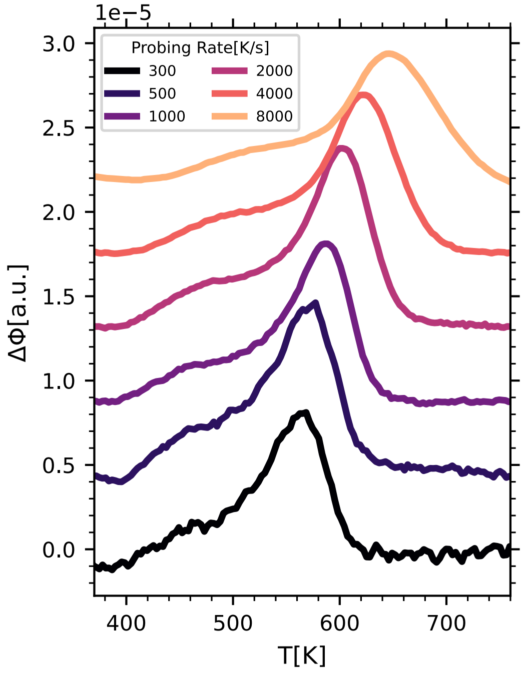

Figure 5. Effect of probing rate on the calorimetric trace of irradiated glasses

Differential thermograms of glasses quenched at 10 K s^{-1}, irradiated at room temperature for 100 s, and probed at the probing rates *r*, listed in the legend. For each probing rate, the differential trace was computed with respect to the trace of the glass quenched at 10 K s^{-1} and then directly probed at the rate *r*. For clarity, the curves have been normalized to the absolute value of the probing rate and vertically shifted by a constant.

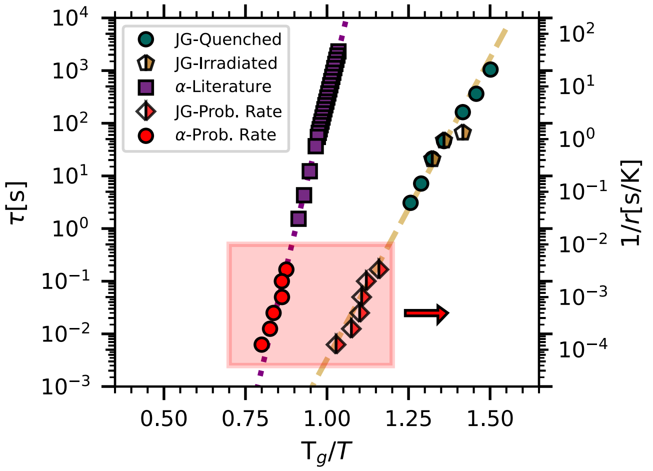

Figure 6. Activation plot for $GeSe_3$

Left *y* axis: enthalpy relaxation times for the low-temperature tail/shoulder for glasses quenched at 20,000 K s^{-1} (green circles) and glasses quenched at 10 K s^{-1} and then irradiated at room temperature for 150 s (half-filled golden pentagons). The probing rate was 2,000 K s^{-1} in all cases. Literature data for the structural relaxation time (purple squares) are also reported.[38] Right *y* axis: inverse probing rate as a function of the inverse T_g-scaled temperature at which (1) the enthalpy recovery peak in the thermograms and (2) the exothermic shoulder in the differential thermograms show the largest slope (red-filled circles and half-filled rhombuses, respectively). The shaded area emphasizes all data corresponding to the right axis to enhance readability. The dotted and dashed lines are linear fits to the literature data for the structural relaxation and to the enthalpy relaxation data for the quenched glasses, respectively. The right *y* axis is shifted vertically by a factor of 50 with respect to the left *y* axis in order to match the data obtained as a function of the probing rate (right *y* axis) with the extrapolation of the structural relaxation and the enthalpy relaxation times (left *y* axis).

The obtained values of T_{md} for the irradiated glasses will be discussed in the following together with the results of the previous two experiments.

Activation plot for the $GeSe_3$ glass

It is now time to bring together the results of the different experiments described so far and to assess the emerging picture.

In Figure 6 (left *y* axis), the enthalpy relaxation times discussed in the previous paragraph are reported as a function of the inverse of the T_g-scaled annealing temperature for the two sets of glasses (see inset legend). For comparison and with reference to the same axis, literature data for the structural relaxation time (ratio of shear viscosity to shear elastic modulus) are also reported.[38] It is interesting to observe that the enthalpy relaxation times for both sets of glasses (1) are clearly different, in terms of both absolute values and slope, from those corresponding to the structural relaxation, and (2) are consistent with each other within error bars, strongly suggesting a common origin for both of them. Moreover, their temperature dependence suggests an activated process described by the classical Arrhenius relation

$$\tau_a = \tau_0 \exp[\Delta E / (k_B T)], \quad \text{(Equation 3)}$$

where ΔE is an activation energy and τ_0 is a constant. Fitting this expression to the enthalpy relaxation time data for the quenched glasses (dashed line in Figure 6) provides the results $\Delta E = 1.01 \pm 0.02$ eV and $\tau_0 = 0.5 \pm 0.4$ ps. In particular, the obtained value for ΔE corresponds to an activation energy of $(24 \pm 1)RT_g$, *R* being the perfect gas constant and T_g being 498 K.[39] This is consistent with the value commonly associated with the JG relaxation for most glasses[15,40] and is assumed to be the fingerprint of such a relaxation. Therefore, we can associate the low-temperature exothermic contribution in the differential thermograms of both thermally quenched and irradiated glasses with the secondary relaxation process despite the differences in shape between the thermograms of the two sets of isochemical glasses. We will further discuss this point in the following. Moreover, the value obtained for τ_0 is of the order of magnitude expected for the inverse of an attempt-to-escape rate, i.e., for a vibrational frequency.

Figure 6 also shows, with reference to the right *y* axis, the dependence of the inverse probing rate as a function of the inverse T_g-scaled temperature where (1) the enthalpy recovery peak in the thermograms (red-filled circles) and (2) the low-temperature exothermic component reported in Figure 5 (half-filled red rhombuses) show the maximum derivative (see Figure S5 for more details). Remarkably, the slopes of these data are compatible, in turn, with the slope of the structural relaxation and with the slope of the data corresponding to the enthalpy

CellPress
OPEN ACCESS

relaxation time. This observation further confirms the assignment of the low-temperature exothermic contribution to the JG relaxation process. Moreover, if we consider the absolute value of the probing rates, we can expect the structural and secondary relaxation times to be proportional to the inverse probing rate, similarly to the well-known proportionality between the structural relaxation time and the inverse cooling rate predicted by the Frenkel-Kobeko-Reiner relation[41,42]:

$$r\tau_\alpha = C, \quad \text{(Equation 4)}$$

where τ_α is the structural relaxation time, r is the quenching rate, and C is a constant. In fact, the right y axis in Figure 6 was shifted vertically by a factor of 50 with respect to the left y axis in order to match the data obtained as a function of the probing rate with the extrapolation of the data obtained from the structural relaxation and the enthalpy relaxation times for the quenched glasses. The value that we obtain for the constant C in Equation 4 (50 K) is similar to those obtained in previous studies focused on the structural relaxation.[42,43] Interestingly, a common value for the constant C seems here to be required to match the datasets corresponding to both the structural and the JG relaxation.

DISCUSSION

Irradiation rejuvenates the glass, driving it to higher-enthalpy states characteristic of glass formed at high cooling rates. This observation, which is a well-known fact in general terms,[6] is here investigated systematically and quantitatively for the case of X-ray irradiation. It is confirmed that X-ray irradiation promotes rejuvenation,[34] but only up to a limiting and not extreme level that has been associated with the yield point of the glass,[33] and that it is iso-enthalpic, for the $GeSe_3$ case, with the glass state reached by quenching at $\sim 10^4$ K s^{-1}.[34] However, in general, iso-enthalpic glasses reached by quenching and irradiation are different glasses with different calorimetric traces and, as we argue in the following, these differences can be interpreted in terms of a different heterogeneity of the corresponding glasses.

The quenched glass in fact inherits its heterogeneity from the supercooled liquid. During quenching, slow-relaxing regions are trapped in states of high enthalpy. Upon heating, these regions relax to lower-enthalpy states before reaching T_g, giving rise to the exothermic, lower-temperature component observed in the calorimetric traces. This is similar to what has been observed and reported for a number of different hyperquenched glasses.[35] In irradiated glasses, however, regions with higher enthalpy (defects) are essentially a consequence of the irradiation process; they also relax upon heating at temperatures below T_g. We have shown here that these defect regions and the higher-enthalpy regions characteristic of the liquid state and trapped in the glass by quenching both relax via a JG relaxation process. In fact, (1) the exothermic component in the FDSC traces appears in the same temperature range below T_g for both quenched and irradiated glasses, and in both cases it increases in strength for higher-enthalpy states; and (2) the enthalpy relaxation times measured in annealing experiments for both quenched and irradiated glasses, and also in experiments changing the probing rate for irradiated glasses, provide consistent results. In particular, the activation energy extracted from the temperature dependence of these characteristic times is the same for both quenched and irradiated glasses and consistent with the one accepted to correspond to the JG relaxation process, i.e., $\approx 26RT_g$.[15,40] Therefore, it seems reasonable to associate the lower-temperature component present in the calorimetric traces of both the quenched and irradiated glasses with the JG relaxation process.

It is interesting to observe that, upon thermal annealing of hyperquenched glasses, a low-temperature endothermic peak often appears on the low-temperature side of the exothermic signal associated with the JG relaxation. This endothermic peak is also known as the shadow glass transition.[35] However, in the glasses investigated in this work, no such endothermic peak is observed. This is likely due to the fact that $GeSe_3$ is a strong glass, and it has been reported that the shadow glass transition does not appear in strong glasses.[36]

The differences in the calorimetric thermograms of the quenched and irradiated glasses can be explained in connection with the different heterogeneity present in these two kinds of glasses. As noted earlier, the increase in enthalpy as a function of irradiation time that is observed in irradiated glasses before reaching the yielding limit appears as a two-step process, in contrast to the more continuous increase for quenched glasses as a function of quenching rate. In the latter case, it is reasonable to imagine that the increase in the number of loosely connected regions for higher quenching rates is inherited by a liquid at higher temperature and is therefore strictly related to the network distortions trapped in the glass. In irradiated glasses, instead, X-rays first promote an increasing number of defects in an almost intact network. This could be the reason for the shoulder shape rather than tail shape for the calorimetric JG relaxation signal. Only later, when the number of defect states is so high that it induces instability in the network, does the network start to relax until eventually yielding. This second route then promotes a much more heterogeneous structure than the first one, at least until when the network relaxes and the yielding point is reached. We note that this picture is fully qualitatively consistent with recent theoretical analyses of glasses exposed to radiation.[44]

The connection that we make here between the islands of mobility usually associated with the JG relaxation process and the defect regions is particularly interesting, as defects in chalcogenide glasses have been studied for a long time.[45] Chalcogenide glasses are characterized by a particular class of defects called valence alternation pairs (VAPs)[46]: for example, in the case of amorphous Se, they correspond to a couple composed of a positively charged ion in a 3-fold configuration and a negatively charged ion in a 1-fold coordinated configuration. Irradiation increases the number of VAP defects, giving rise to a number of light-induced phenomena such as photodarkening, i.e., a redshift of the band edge.[47] As a matter of fact, the activation energy for the JG relaxation in $GeSe_3$ that we find here corresponds to energy barriers of $\sim$1 eV. Similar activation energies around 0.8–0.9 eV have been reported in amorphous Se for the relaxation of photodarkening[48,49] and are also suggested for relaxation of VAP defects.[45,49] Therefore, it seems reasonable to associate the defective structures in $GeSe_3$ related to VAP defects with the

loosely connected regions that relax via JG relaxation processes. This association is further supported by the following observation.

We have recently shown[34] that X-ray irradiation induces a yielding transition in the very same glass studied here, $GeSe_3$, at doses (irradiation times) comparable to those discussed here. Microscopically, yielding—and more generally, plasticity—occurs in glasses via the rearrangement of regions involving several tens of atoms known as shear transformation zones[50] and is observed in numerical simulations[24] and experimentally in colloidal glasses.[51] These shear transformation zones are essentially groups of atoms within a relatively loosely packed region that undergo a plastic distortion from one configuration to another while crossing an energy barrier, similarly to the previously mentioned relaxation mechanism of VAP defects in chalcogenides. An estimation of the energy barrier relevant for shear transformation zones has in fact been shown to be correlated with the activation energy for JG relaxation.[14]

X-ray irradiation, then, seems to be a tool to increase the JG relaxation strength also in rather strong glasses where it is known to be usually very weak,[52] in agreement with recent theoretical predictions concerning the effects of irradiation in amorphous materials.[44] This is consistent with the fact that plastic deformation, the most common method used to rejuvenate glasses, has been reported to enhance the strength of JG relaxation.[53,54] Conversely, more stable glasses show a reduction of this strength,[9] suggesting a clear evolutionary pattern.

It would clearly be interesting to confirm the present results by mechanical spectroscopy experiments, which have substantially contributed to our current understanding of the JG process in glasses.[14] Unfortunately, the irradiated samples we can produce at the moment have sizes in the ~10 μm range and are thus too small for these experiments. FDSC is, conversely, perfectly suited for this sample size, which is the reason why here we have focused our attention on this technique.

In conclusion, we have provided here a novel guideline for further research to develop glasses in high-enthalpy states with high JG relaxation strengths through the control of structural heterogeneity. In particular, we have shown that iso-enthalpic glasses prepared by melt-quenching or X-ray irradiation show different calorimetric traces and are therefore distinct glasses. The main difference is in the better defined shape, in the calorimetric traces of the irradiated glasses before yielding, of an exothermic contribution, which appears at temperatures lower than T_g and which we can associate with the JG relaxation process. Despite this difference, which can be traced to a different structural heterogeneity, both quenched and irradiated glasses show the calorimetric signature of the JG relaxation, and the corresponding relaxation parameters, for both sets of glasses, are mutually consistent within error bars. In particular, this means that the complex structural processes that take place in a glass upon irradiation can be associated with growing strength of the JG relaxation, which takes place around the defects generated by X-ray irradiation and behaves as a plastic event: their number grows until the structure relaxes and the yield point is reached. From a fundamental perspective, the present results strengthen the interpretation of the JG relaxation as being due to loosely connected or defect regions. From a more applied perspective, given that the secondary relaxation is directly related to several mechanical properties of glasses,[14] we offer here an alternative pathway to the quenching rate to prepare glass states with tailored mechanical properties.

METHODS

We focus here on $GeSe_3$ glass, a well-known, simple binary compound representative of the class of chalcogenide glasses.[47] This glass has been at the focus of previous structural studies,[55] and some effects of X-ray irradiation have been studied in a previous investigation.[34] The $GeSe_3$ samples studied here were prepared starting from pure elements as detailed in Baglioni et al.[34] Small pieces were taken from the bulk sample and mounted on the membrane chip (UFH 1 sensor) of a Flash DSC 2+ calorimeter from Mettler Toledo purged with nitrogen at 20 mL s^{-1}. The sample was melted on the chip to ensure a good thermal contact and then cooled down to room temperature at the desired cooling rate. After melting, the lateral extension of the sample size on the chip was ~35 μm, and its thickness was ~30 μm.

The calorimeter was used to prepare glasses by quenching the undercooled liquid from 773 K to room temperature at different cooling rates in the range 10–2 × 10^4 K s^{-1}. The FDSC probing measurements were performed by heating each glass from room temperature to 773 K at a rate of 2,000 K s^{-1}, followed by cooling back the sample to room temperature at the same rate. The whole series of measurements was repeated more than once to verify reproducibility. Whenever useful, the baseline-corrected calorimetric traces were converted to absolute units. Since the mass of the samples melted on the calorimetric chip is too small to be directly measured (it is on the order of 10^2 ng), this was done using known specific heat data in the glass,[56] as discussed in Baglioni et al.[34]

The FDSC measurements for a glass quenched to room temperature from the melt at 773 K at a rate of 10 K s^{-1} and irradiated for different periods of time were carried out using the setup discussed in Martinelli et al.[57] In particular, we used the Flash DSC 2+ calorimeter integrated in the coherence beamline P10 at Petra III (DESY, Hamburg).[58] The X-ray photon energy was set at 8.1 keV (λ = 1.53 Å), and the beam was focused to a spot of 98 × 93 μm full width at half maximum (*H* × *V*). A beam energy with an attenuation length in the material longer than the sample thickness and with a beam spot larger than the lateral extension of the sample is necessary to guarantee a uniform and complete irradiation of the glass and to study the corresponding photoinduced thermodynamic variation. The X-ray beam intensity was 9 × 11 ph s^{-1} corresponding, for our samples, to a dose rate of ~0.4 MGy s^{-1}.[34] In these conditions, the temperature rise induced by the X-ray beam, as directly detected by the temperature sensors of the calorimeter, is less than 2 K[34] and thus negligible for the discussion of the results presented here.

The sample on the membrane chip of the calorimeter was kept in a vacuum at room temperature and irradiated with the X-ray beam for periods in the range 0.5–1,800 s. The membrane chip for the FDSC scans has two identical cells, as the calorimetric measurements are based on a differential approach. Following each irradiation of the sample, the reference side of the membrane chip was also irradiated for the same amount of time,

leveraging the differential method to correct for any possible X-ray-induced effects on the chip membrane. After each irradiation sequence for the sample and reference, the calorimetric trace was collected by heating the sample to 773 K at 2,000 K s^{-1}. Immediately thereafter, a glass prepared at 10 K s^{-1} was also probed at 2,000 K s^{-1} to establish the baseline and precisely quantify the X-ray-induced modifications. The maximum temperature of each cycle (773 K) is well above the glass transition and was verified to be sufficiently high to completely erase the memory of the previous irradiations.

A series of additional measurements was performed to study enthalpy relaxation during aging below T_g for both fast-quenched glasses and irradiated glasses, following a strategy already used to study other glasses.[15] Moreover, one more set of measurements was collected as a function of the probing rate in the range 300–8,000 K s^{-1} for the glass prepared by cooling at 10 K s^{-1} and then irradiated for 100 s. In all cases, the same measurement scheme as discussed above was adopted.

RESOURCE AVAILABILITY

Lead contact

Requests for further information and resources should be directed to and will be fulfilled by the lead contact, Jacopo Baglioni (jacopo.baglioni@unipd.it).

Materials availability

This study did not generate new materials.

Data and code availability

Any additional information required to reanalyze the data reported in this paper is available from the lead contact upon request.

ACKNOWLEDGMENTS

This research was carried out at beamline P10 (long-term project II-20210011EC) at DESY, a member of the Helmholtz Association (HGF). J.B. acknowledges support from the Centre for Molecular Water Science (CMWS) in an Early Science Project. The research leading to this result has been supported by the project GLAXES ERC-2021-ADG (grant agreement no. 101053167) funded by the European Union. Dr. C. Scian from the Physics and Astronomy Department of the University of Padova is gratefully acknowledged for his support in the preparation of the samples.

AUTHOR CONTRIBUTIONS

Conceptualization, J.B. and G.M.; methodology, J.B. and G.M.; formal analysis, J.B.; investigation, J.B., A.M., P.S., F.D., L.P., F.W., M.S., and G.M; software, J.B. , writing – original draft, J.B. and G.M.; writing – review & editing, J.B., A.M., P.S., F.D., L.P., M.U., F.W., M.S., and G.M.; visualization, J.B.; funding acquisition, G.M.; resources, J.B. and L.P.; supervision, G.M.

DECLARATION OF INTERESTS

The authors declare no competing interests.

SUPPLEMENTAL INFORMATION

Supplemental information can be found online at https://doi.org/10.1016/j.newton.2025.100338.

Received: June 9, 2025
Revised: September 15, 2025
Accepted: November 18, 2025
Published: December 18, 2025

REFERENCES

1. Debenedetti, P.G., and Stillinger, F.H. (2001). Supercooled liquids and the glass transition. Nature *410*, 259–267. https://doi.org/10.1038/35065704.
2. Jiang, Y., Ji, Y., Liu, H., Liu, D., Wang, L., Jiang, C., Yang, Y., and Chen, D. (2012). Insights into effects of thermal annealing on optical properties of SiO_2 films. In 6th International Symposium on Advanced Optical Manufacturing and Testing Technologies: Advanced Optical Manufacturing Technologies, L. Yang, E. Ruch, and S. Li, eds. (SPIE). https://doi.org/10.1117/12.974980.
3. Pan, J., Wang, Y.X., Guo, Q., Zhang, D., Greer, A.L., and Li, Y. (2018). Extreme rejuvenation and softening in a bulk metallic glass. Nat. Commun. *9*, 560. https://doi.org/10.1038/s41467-018-02943-4.
4. Mota, R.M.O., Lund, E.T., Sohn, S., Browne, D.J., Hofmann, D.C., Curtarolo, S., van de Walle, A., and Schroers, J. (2021). Enhancing ductility in bulk metallic glasses by straining during cooling. Commun. Mater. *2*, 23. https://doi.org/10.1038/s43246-021-00127-0.
5. Qiao, A., Wang, P., Tao, H., and Yue, Y. (2018). Sub-Tg enthalpy relaxation in milled and quenched As_2S_3 glasses. J. Non-Cryst. Solids *500*, 225–230. https://doi.org/10.1016/j.jnoncrysol.2018.08.003.
6. Sun, Y., Concustell, A., and Greer, A.L. (2016). Thermomechanical processing of metallic glasses: extending the range of the glassy state. Nat. Rev. Mater. *1*, 16039. https://doi.org/10.1038/natrevmats.2016.39.
7. Khademorezaian, S., Tomut, M., Peterlechner, M., da Silva Pinto, M.W., Rösner, H., Divinski, S., and Wilde, G. (2024). Extreme rejuvenation of a bulk metallic glass at the nanoscale by swift heavy ion irradiation. J. Alloys Compd. *980*, 173571. https://doi.org/10.1016/j.jallcom.2024.173571.
8. Ichitsubo, T., Matsubara, E., Yamamoto, T., Chen, H.S., Nishiyama, N., Saida, J., and Anazawa, K. (2005). Microstructure of fragile metallic glasses inferred from ultrasound-accelerated crystallization in Pd-based metallic glasses. Phys. Rev. Lett. *95*, 245501. https://doi.org/10.1103/PhysRevLett.95.245501.
9. Yu, H.B., Tylinski, M., Guiseppi-Elie, A., Ediger, M.D., and Richert, R. (2015). Suppression of β relaxation in vapor-deposited ultrastable glasses. Phys. Rev. Lett. *115*, 185501. https://doi.org/10.1103/PhysRevLett.115.185501.
10. Zhu, F., Nguyen, H.K., Song, S.X., Aji, D.P.B., Hirata, A., Wang, H., Nakajima, K., and Chen, M.W. (2016). Intrinsic correlation between β-relaxation and spatial heterogeneity in a metallic glass. Nat. Commun. *7*, 11516. https://doi.org/10.1038/ncomms11516.
11. Johari, G.P., and Goldstein, M. (1970). Viscous liquids and the glass transition. II. secondary relaxations in glasses of rigid molecules. J. Chem. Phys. *53*, 2372–2388. https://doi.org/10.1063/1.1674335.
12. Wang, Q., Zhang, S.T., Yang, Y., Dong, Y.D., Liu, C.T., and Lu, J. (2015). Unusual fast secondary relaxation in metallic glass. Nat. Commun. *6*, 7876. https://doi.org/10.1038/ncomms8876.
13. Lunkenheimer, P., Schneider, U., Brand, R., and Loid, A. (2000). Glassy dynamics. Contemp. Phys. *41*, 15–36. https://doi.org/10.1080/001075100181259.
14. Yu, H.B., Wang, W.H., Bai, H.Y., Wu, Y., and Chen, M.W. (2010). Relating activation of shear transformation zones to β-relaxations in metallic glasses. Phys. Rev. B *81*, 220201–R. https://doi.org/10.1103/PhysRevB.81.220201.
15. Hu, L., and Yue, Y. (2009). Secondary relaxation in metallic glass-formers: its correlation with the genuine Johari-Goldstein relaxation. J. Phys. Chem. C *113*, 15001–15006. https://doi.org/10.1021/jp903777f.
16. Yang, Q., Peng, S.X., Wang, Z., and Yu, H.B. (2020). Shadow glass transition as a thermodynamic signature of β-relaxation in hyper-quenched metallic glasses. Natl. Sci. Rev. *7*, 1896–1905. https://doi.org/10.1093/nsr/nwaa100.
17. Cangialosi, D. (2024). Physical aging and vitrification in polymers and other glasses: Complex behavior and size effects. J. Polym. Sci. *62*, 1952–1974. https://doi.org/10.1002/pol.20230850.

18. Kudlik, A., Benkhof, S., Blochowicz, T., Tschirwitz, C., and Rössler, E. (1999). The dielectric response of simple organic glass formers. J. Mol. Struct. *479*, 201–218. https://doi.org/10.1016/s0022-2860(98)00871-0.
19. Yu, H.B., Wang, W.H., and Samwer, K. (2013). The β-relaxation in metallic glasses: an overview. Mater. Today *16*, 183–191. https://doi.org/10.1016/j.mattod.2013.05.002.
20. Xiao, C., Jho, J.Y., and Yee, A.F. (1994). Correlation between the shear yielding behavior and secondary relaxations of bisphenol A polycarbonate and related copolymers. Macromolecules *27*, 2761–2768. https://doi.org/10.1021/MA00088A017.
21. Ngai, K.L., Capaccioli, S., Ancherbak, S., and Shinyashiki, N. (2011). Resolving the ambiguity of the dynamics of water and clarifying its role in hydrated proteins. Philos. Mag. *91*, 1809–1835. https://doi.org/10.1080/14786435.2010.523716.
22. Chen, L.P., Yee, A.F., and Moskala, E.J. (1999). The molecular basis for the relationship between the secondary relaxation and mechanical properties of a series of polyester copolymer glasses. Macromolecules *32*, 5944–5955. https://doi.org/10.1021/ma981363a.
23. Ngai, K.L., Wang, L.M., Liu, R., and Wang, W.H. (2014). Microscopic dynamics perspective on the relationship between Poisson's ratio and ductility of metallic glasses. J. Chem. Phys. *140*, 044511. https://doi.org/10.1063/1.4862822.
24. Falk, M.L., and Langer, J.S. (1998). Dynamics of viscoplastic deformation in amorphous solids. Phys. Rev. E *57*, 7192–7205. https://doi.org/10.1103/PhysRevE.57.7192.
25. Berthier, L., Biroli, G., Bouchaud, J.P., Cipelletti, L., and van Saarloos, W. (2011). Dynamical Heterogeneities in Glasses, Colloids, and Granular Media (Oxford University Press). https://doi.org/10.1093/acprof:oso/9780199691470.001.0001.
26. Caporaletti, F., Capaccioli, S., Valenti, S., Mikolasek, M., Chumakov, A.I., and Monaco, G. (2021). Experimental evidence of mosaic structure in strongly supercooled molecular liquids. Nat. Commun. *12*, 1867. https://doi.org/10.1038/s41467-021-22154-8.
27. Dyre, J.C. (2024). Solid-that-flows picture of glass-forming liquids. J. Phys. Chem. Lett. *15*, 1603–1617. https://doi.org/10.1021/acs.jpclett.3c03308.
28. Gao, L., Yu, H.B., Schrøder, T.B., and Dyre, J.C. (2025). Unified percolation scenario for the α and β processes in simple glass formers. Nat. Phys. *21*, 471–479. https://doi.org/10.1038/s41567-024-02762-z.
29. Stillinger, F.H. (1995). A topographic view of supercooled liquids and glass formation. Science *267*, 1935–1939. https://doi.org/10.1126/science.267.5206.1935.
30. Heuer, A. (2008). Exploring the potential energy landscape of glass-forming systems: from inherent structures via metabasins to macroscopic transport. J. Phys. Condens. Matter *20*, 373101. https://doi.org/10.1088/0953-8984/20/37/373101.
31. Shiraishi, K., Mizuno, H., and Ikeda, A. (2023). Johari–Goldstein β-relaxation in glassy dynamics originates from two-scale energy landscape. Proc. Natl. Acad. Sci. USA *120*, e2215153120. https://doi.org/10.1073/pnas.2215153120.
32. Dallari, F., Martinelli, A., Caporaletti, F., Sprung, M., Baldi, G., and Monaco, G. (2023). Stochastic atomic acceleration during the x-ray-induced fluidization of a silica glass. Proc. Natl. Acad. Sci. USA *120*, e2213182120. https://doi.org/10.1073/pnas.2213182120.
33. Martinelli, A., Caporaletti, F., Dallari, F., Sprung, M., Westermeier, F., Baldi, G., and Monaco, G. (2023). Reaching the yield point of a glass during x-ray irradiation. Phys. Rev. X *13*, 041031. https://doi.org/10.1103/PhysRevX.13.041031.
34. Baglioni, J., Martinelli, A., Sun, P., Dallari, F., Westermeier, F., Sprung, M., Grübel, G., and Monaco, G. (2024). Uniqueness of glasses prepared via x-ray induced yielding. Rep. Prog. Phys. *87*, 120503. https://doi.org/10.1088/1361-6633/ad91df.
35. Yue, Y. (2022). Revealing the nature of glass by the hyperquenching-annealing-calorimetry approach. J. Non-Cryst. Solids X *14*, 100099. https://doi.org/10.1016/j.nocx.2022.100099.
36. Hu, L., and Yue, Y. (2008). Secondary relaxation behavior in a strong glass. J. Phys. Chem. B *112*, 9053–9057. https://doi.org/10.1021/jp711696p.
37. Ritland, H.N. (1956). Limitations of the fictive temperature concept. J. Am. Ceram. Soc. *39*, 403–406. https://doi.org/10.1111/j.1151-2916.1956.tb15613.x.
38. Zeidler, A., Salmon, P.S., Whittaker, D.A.J., Pizzey, K.J., and Hannon, A.C. (2017). Topological ordering and viscosity in the glass-forming Ge–Se system: The search for a structural or dynamical signature of the intermediate phase. Front. Mater. *4*, 32. https://doi.org/10.3389/fmats.2017.00032.
39. Gueguen, Y., Rouxel, T., Gadaud, P., Bernard, C., Keryvin, V., and Sangleboeuf, J.C. (2011). High-temperature elasticity and viscosity of Ge_xSe_{1-x} glasses in the transition range. Phys. Rev. B *84*, 064201. https://doi.org/10.1103/PhysRevB.84.064201.
40. Ngai, K.L., and Capaccioli, S. (2004). Relation between the activation energy of the Johari-Goldstein β-relaxation and Tg of glass formers. Phys. Rev. E *69*, 031501.https://doi.org/10.1103/PhysRevE.69.031501.
41. Gutzow, I., and Schmelzer, J. (1995). The Vitreous State (Berlin: Springer-Verlag). https://doi.org/10.1007/978-3-662-03187-2.
42. Schawe, J.E.K. (2014). Vitrification in a wide cooling rate range: the relations between cooling rate, relaxation time, transition width, and fragility. J. Chem. Phys. *141*, 184905. https://doi.org/10.1063/1.4900961.
43. Di Lisio, V., Stavropoulou, V.M., and Cangialosi, D. (2023). Physical aging in molecular glasses beyond the α-relaxation. J. Chem. Phys., 159. https://doi.org/10.1063/5.0157994.
44. Lubchenko, V., and Wolynes, P.G. (2020). Photon activation of glassy dynamics: A mechanism for photoinduced fluidization, aging, and information storage in amorphous materials. J. Phys. Chem. B *124*, 8434–8453. https://doi.org/10.1021/acs.jpcb.0c06515.
45. Tanaka, K. (1983). Mechanisms of photodarkening in amorphous chalcogenides. J. Non-Cryst. Solids *12*, 59. https://doi.org/10.1016/0022-3093(83)90320-4.
46. Kastner, M., Adler, D., and Fritzsche, H. (1976). Valence-alternation model for localized gap states in lone-pair semiconductors. Phys. Rev. Lett. *37*, 1504–1507. https://doi.org/10.1103/PhysRevLett.37.1504.
47. Popescu, M.A. (2000). Non-Crystalline Chalcogenides (Kluwer Academic Publishers). https://doi.org/10.1007/0-306-47129-9.
48. Reznik, A., Lui, B.J.M., Rowlands, J.A., Baranovskii, S.D., Rubel, O., Lyubin, V., Klebanov, M., Kasap, S.O., Ohkawa, Y., Matsubara, T., et al. (2006). Kinetics of the photostructural changes in a-Se films. J. Appl. Phys. *100*, 113506. https://doi.org/10.1063/1.2372316.
49. Berashevich, J., Mishchenko, A., and Reznik, A. (2014). Two-step photo-excitation mechanism in amorphous Se. Phys. Rev. Appl. *1*, 034008. https://doi.org/10.1103/PhysRevApplied.1.034008.
50. Falk, M.L., and Langer, J.S. (2011). Deformation and failure of amorphous, solid-like materials. Annu. Rev. Condens. Matter Phys. *2*, 353–373. https://doi.org/10.1146/annurev-conmatphys-062910-140452.
51. Schall, P., Weitz, D.A., and Spaepen, F. (2007). Structural rearrangements that govern flow in colloidal glasses. Science *318*, 1895–1899. https://doi.org/10.1126/science.1149308.
52. Peng, S.X., Cheng, Y., Pries, J., Wei, S., Yu, H.B., and Wuttig, M. (2020). Uncovering β-relaxations in amorphous phase-change materials. Sci. Adv. *6*, eaay6726. https://doi.org/10.1126/sciadv.aay6726.
53. Cao, Q.P., Li, J.F., Zhou, Y.H., Horsewell, A., and Jiang, J.Z. (2005). Free-volume evolution and its temperature dependence during rolling of $Cu_{60}Zr_{20}Ti_{20}$ bulk metallic glass. Appl. Phys. Lett. *87*, 101901. https://doi.org/10.1063/1.2037858.
54. Méar, F., Lenk, B., Zhang, Y., and Greer, A. (2008). Structural relaxation in heavily cold-worked metallic glass. Scr. Mater. *59*, 1243–1246. https://doi.org/10.1016/j.scriptamat.2008.08.023.

CellPress
OPEN ACCESS

55. Rowlands, R.F., Zeidler, A., Fischer, H.E., and Salmon, P.S. (2019). Structure of the intermediate phase glasses $GeSe_3$ and $GeSe_4$: The deployment of neutron diffraction with isotope substitution. Front. Mater. *6*, 133. https://doi.org/10.3389/fmats.2019.00133.

56. Borisova, Z.U. (1981). Glassy Semiconductors (Springer US). https://doi.org/10.1007/978-1-4757-0851-6.

57. Martinelli, A., Baglioni, J., Sun, P., Dallari, F., Pineda, E., Duan, Y., Spitzbart-Silberer, T., Westermeier, F., Sprung, M., and Monaco, G. (2024). A new experimental setup for combined fast differential scanning calorimetry and x-ray photon correlation spectroscopy. J. Synchrotron Radiat. *31*, 557–565. https://doi.org/10.1107/s1600577524002510.

58. DESY(2023). PETRA III P10 beamline photon-science.desy.de/facilities/petra_iii/beamlines/p10_coherence_applications.

NEWTON, Volume *2*

Supplemental information

Johari-Goldstein relaxation in quenched and irradiated chalcogenide glasses

Jacopo Baglioni, Alessandro Martinelli, Peihao Sun, Francesco Dallari, Lara Piemontese, Muhammad Umair, Fabian Westermeier, Michael Sprung, and Giulio Monaco

Note S1 Evaluation of the enthalpy variation in quenched glasses

The enthalpy variation induced by the annealing of thermally quenched glasses is calculated from the thermograms as shown in Fig. S1. The enthalpy is calculated integrating the difference between the Maier-Kelley extrapolation (dashed blue line) of the low-temperature values of the thermogram and the calorimetric traces [1].

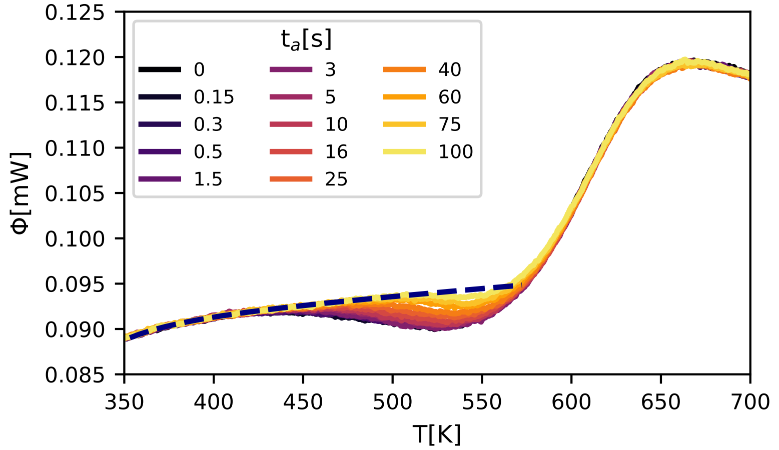

Figure S1: Themograms of a $GeSe_3$ glass quenched at 20 000 K s^{-1} and annealed at 368 K for various time intervals as listed in the legend. The dashed line is the Maier-Kelley extrapolation of the low temperature values of the thermal power.

Note S2 Secondary relaxation strength

Different strategies to separate the contribution of the primary and secondary relaxations in the calorimetric traces can be adopted, the one used in this work is shown in Fig. S2. The differential thermogram across the glass transition region is fitted with a Gaussian curve (black line) and the area below the curve is associated to the structural relaxation, while the remaining enthalpy contribution is assigned to the Johari-Goldstein (JG) relaxation. This method avoids the introduction of a threshold temperature between the two relaxations, thereby reducing the arbitrariness of their separation.

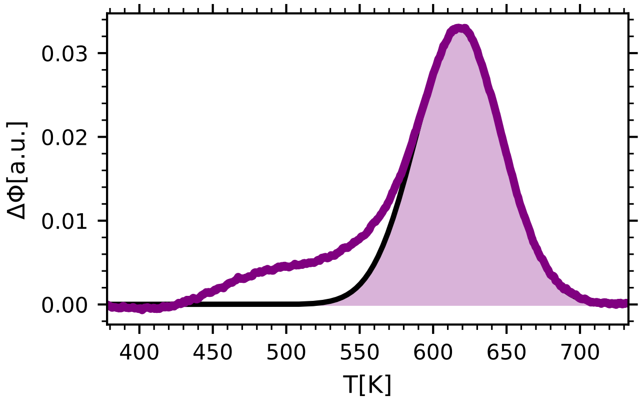

Figure S2: Differential thermogram of an irradiated $GeSe_3$ glass (purple line) and the Gaussian fit to the main peak (black line). The shaded region is proportional to the enthalpy contribution associated with the structural relaxation, while the residual area is associated with the Johari-Goldstein one.

Note S3 Stretched exponential fit to the enthalpy relaxation data

The values of the relaxation time, τ_a, and shape parameter, β_a, obtained from the fit of a stretched exponential function, Eq. 2 in the main text, to the enthalpy relaxation data as a function of the annealing time reported in Fig. 3 and Fig. 4 of the main text are shown in Fig. S3 and Fig. S4 for the thermally quenched and for the irradiated $GeSe_3$ glasses, respectively. The dashed line in panel **A** of Fig. S3 is the best fitting Arrhenius function, Eq. 3 in the main text, to the enthalpy relaxation data. The results obtained from the fit are discussed in the main text. The Arrhenius function with the same parameters is also reported in panel **a** of Fig. S4 to demonstrate compatibility with the enthalpy relaxation data for the irradiated glasses. For what concerns the shape parameters, they show no clear dependence on temperature, and have the average values β_a=1.06 ± 0.01 (0.63 ± 0.08) for the quenched (irradiated) glasses. The lower value of β_a for the irradiated glasses is compatible with the theoretical idea that a broad range of relaxation times in an heterogeneous structure is induced by irradiation [2].

Note S4 Thermograms at different probing rates

The thermograms collected as a function of the probing rate are reported in Fig. S5 **A**. They refer to a glass quenched from the melt to room temperature at $10\,\mathrm{K\,s^{-1}}$, irradiated for 420 s and then probed at various heating rates, r,

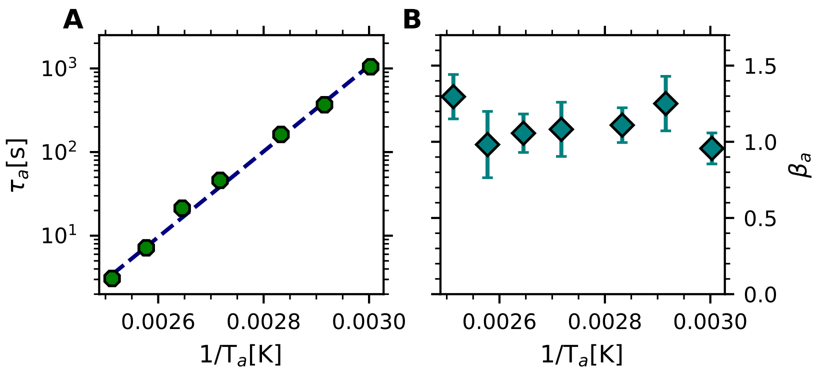

Figure S3: Relaxation time (**A**) and shape factor (**B**) obtained from fitting a stretched exponential function to the enthalpy relaxation data in Fig. 3 in the main text. The dashed line is the best fitting Arrhenius function used to extract the activation energy of the Johari-Goldstein relaxation.

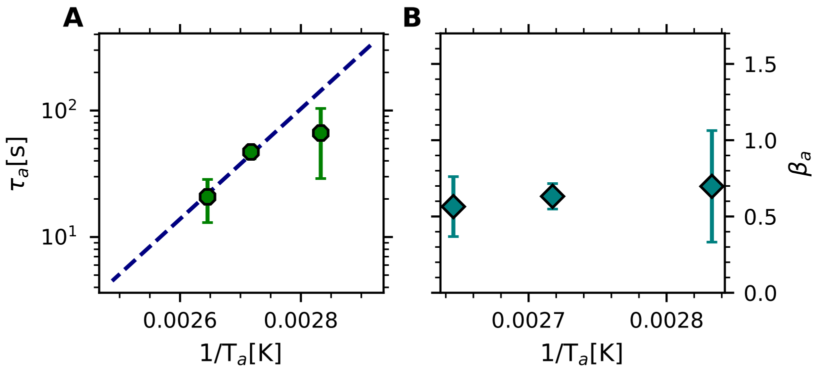

Figure S4: Relaxation time (**A**) and shape factor (**B**) obtained from fitting a stretched exponential function to the enthalpy relaxation data in Fig. 4 in the main text. The dashed line is the Arrhenius function obtained from fitting the enthalpy relaxation data for the quenched glasses and reported in Fig. S3.

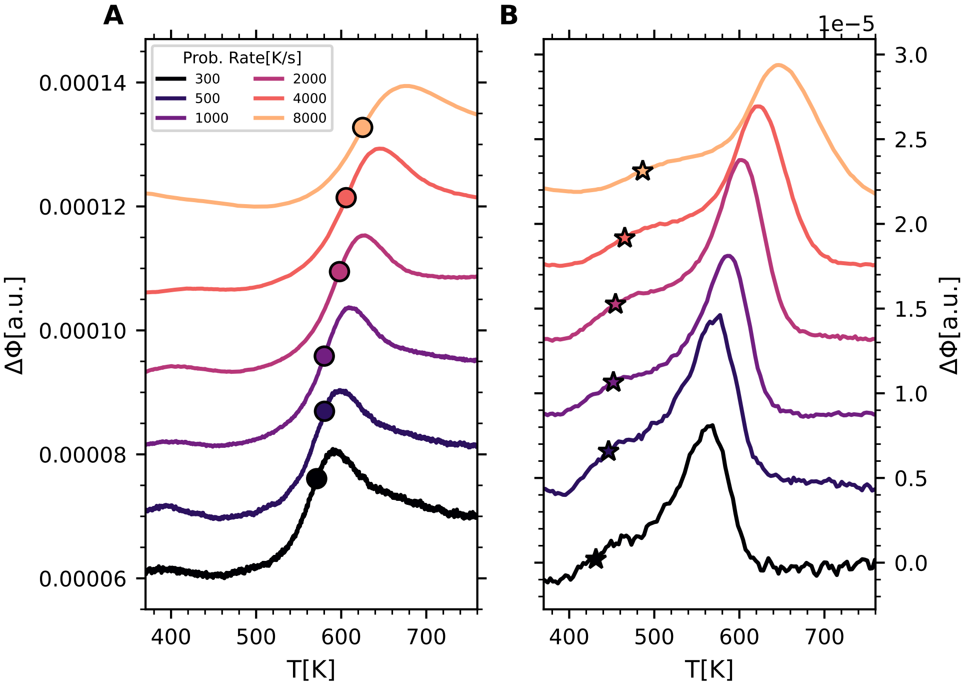

Figure S5: **A** Thermograms measured for $GeSe_3$ glasses irradiated for 100 s at room temperature and probed with different heating rates r (listed in the legend). The curves have been normalized by the absolute value of the rate. The circles mark the points of the maximum derivative of the enthalpy recovery peak. **B** Differential thermograms of the data in **A** computed with reference to the trace of a glass quenched at $10\,\mathrm{K\,s^{-1}}$ and then probed at the rate r. The stars mark the position of the maximum derivative of the exothermic signal associated with the JG relaxation.

ranging from $300\,\mathrm{K\,s^{-1}}$ to $8000\,\mathrm{K\,s^{-1}}$ and listed in the legend. For each probing rate, a differential trace was computed using the trace of a glass quenched at $10\,\mathrm{K\,s^{-1}}$ and then directly probed at the rate r. The obtained differential traces are reported in Fig. S5 **B** and are the same as in Fig. 5 in the main text. The characteristic temperatures of the enthalpy recovery peak (circles, associated to the structural relaxation) and of the exothermic peak appearing in the differential thermograms (stars, associated with the JG relaxation) are also reported in Fig. S5 **A** and **B**, respectively. These characteristic temperatures, which have been used in Fig. 6 in the main text in combination with the probing rates, correspond to the maximum value of the derivative of the enthalpy recovery peak (circles in Fig. S5 **A**) and to the maximum value of the derivative of the exothermic signal in the differential thermograms (stars in Fig. S5 **B**).

Supplemental References

[1] Y.Z. Yue, J.deC. Christiansen, and S.L. Jensen. Determination of the fictive temperature for a hyperquenched glass. *Chemical Physics Letters*, 357(1):20–24, 2002.

[2] Vassiliy Lubchenko and Peter G. Wolynes. Photon activation of glassy dynamics: A mechanism for photoinduced fluidization, aging, and information storage in amorphous materials. *The Journal of Physical Chemistry B*, 124(38):8434–8453, August 2020.
